# Interoceptive abilities facilitate taking another’s spatial perspective

**DOI:** 10.1038/s41598-023-36173-6

**Published:** 2023-06-21

**Authors:** Chiara Baiano, Xavier Job, Louise P. Kirsch, Malika Auvray

**Affiliations:** 1 Department of Psychology, University of Campania Luigi Vanvitelli, 81100 Caserta, Italy; 2 Department of Neuroscience, Karolinska Institute, Stockholm, Sweden; 3 Integrative Neuroscience and Cognition Center, CNRS, UMR 8002, Université Paris Cité, 45 Rue des Saints Pères, 75006 Paris, France; 4 Institut des Systèmes Intelligents et de Robotique, ISIR, CNRS, Sorbonne Université, 4 Place Jussieu, 75005 Paris, France

**Keywords:** Perception, Human behaviour

## Abstract

Information can be perceived from a multiplicity of spatial perspectives, which is central to effectively understanding and interacting with our environment and other people. Interoception, the sense of the physiological state of our body, is also a fundamental component contributing to our perception. However, whether the perception of our inner body signals influences our ability to adopt and flexibly change between different spatial perspectives remains poorly understood. To investigate this, 90 participants completed tasks assessing multiple dimensions of interoception (interoceptive sensibility, cardiac interoceptive accuracy and awareness) and the Graphesthesia task to assess tactile spatial perspective-taking and its flexibility. The results revealed that higher cardiac interoceptive awareness is associated with greater consistency in adopting a perspective decentred from the self. Second, higher cardiac interoceptive accuracy was associated with slower and less accurate performance in switching from a decentred to an egocentred perspective. These results show that interoceptive abilities facilitate decentred spatial perspective-taking, likely reflecting stronger perceived boundaries between internal states and the external world.

## Introduction

Perceiving the world from our first-person perspective is necessarily grounded in our bodies, but what happens when we perceive the world from someone else’s perspective? Does perception remain grounded in our body? In other words, is it influenced by the way we perceive our internal signals? Indeed, it has been acknowledged that internal signals such as cardiac inputs influence cognition at several levels^1,2^. Moreover, the ability to perceive our internal signals (i.e. interoception) has been shown to interact with several cognitive processes^3,4^. However, the extent to which interoception influences spatial perspective-taking, i.e., to the ability to mentally displace the self into a new position and orientation, remains largely unknown.

Interoception refers to the conscious and unconscious processing of signals originating from within the body by the nervous system, which provides moment-by-moment mapping representing the physiological state of the body^5,6^. At a conscious level, Garfinkel et al.^7^ distinguished three partially dissociable dimensions for the assessment of interoceptive abilities: (i) interoceptive accuracy (performance on objective behavioural tests of heartbeat detection or discrimination); (ii) interoceptive sensibility (subjective measures assessed using questionnaires); (iii) interoceptive awareness (metacognitive awareness of interoceptive accuracy).

Perspective-taking is a multidimensional construct referring to the ability to take another person’s point of view. It is often characterised along cognitive, affective, and spatial dimensions that are closely related to each other^8–10^. Spatial perspective-taking, corresponding to the ability to understand the visuo-spatial experience of another agent^11^, has been described as an embodied process, grounded in the internal representations of our body and requiring cognitive transformations of one’s own visuospatial viewpoint into another’s location and orientation^12,13^. This process is underpinned by proprioceptive representations, requiring information about our body position and posture to understand others’ spatial coordinates^13^.

Recent evidence highlights possible links between interoception and perspective-taking. For instance, studies have identified relationships between interoceptive processing and emotional and cognitive perspective-taking (i.e. empathy and Theory of Mind^14–16^). Moreover, emotional perspective-taking appears to be related to spatial perspective-taking. However, it was not associated with other non-perspective-taking spatial abilities such as mental rotation^17^. In line with this, Erle^18^ found that interoceptive accuracy (quantified by the heartbeat counting task, Schandry^19^) is related to faster and more accurate level-2 visual perspective-taking performance (i.e., the ability to understand how an object is perceived from another point of view). However, a subsequent link between other dimensions of interoception (i.e. interoceptive sensibility and interoceptive awareness) and spatial perspective-taking remains poorly understood (see Baiano et al.^20^ for a review). In addition, no study to date has taken into account the flexibility in spatial perspective taking, i.e. the ability to switch between different spatial points of view.

**Figure 1:**
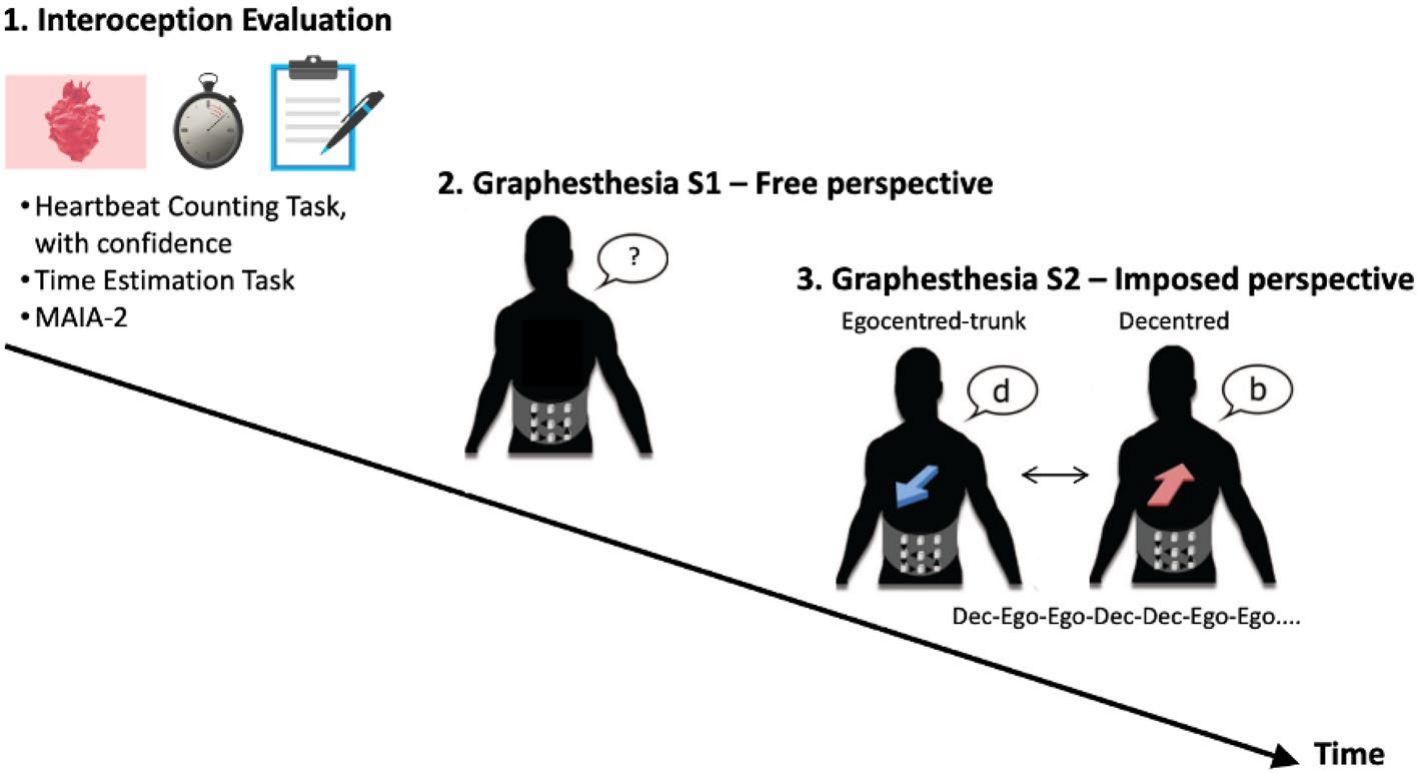
Experimental procedure. First, the participants performed the Heartbeat Counting Task, with confidence ratings on their performance, followed by the Time Estimation Task, and then completed the MAIA-2 (see “Procedure”). Second, the participants performed the Graphesthesia task without any instruction regarding how they should interpret the letters b, d, p, q, to assess which perspective they spontaneously adopted (Graphesthesia session 1, free spatial perspective). Finally, participants performed a second session (S2) of the Graphesthesia task in which they were instructed to take the perspective indicated on the screen (Graphesthesia session 2, imposed switch spatial perspective). Note that the conditions Egocentred-trunk (ego) and Decentred (dec) are displayed here as an example; but for those participants who had a majority of responses with an Egocentred-head perspective in the second part (Natural), the Egocentred-trunk condition was replaced with the Egocentred-head condition. *SPT:* spatial perspective-taking.

To clarify the possible influence of interoceptive abilities on spatial perspective-taking, the study reported here takes advantage of the Graphesthesia task^21^, which provides an ideal tool to investigate the embodied nature of spatial perspective-taking. The task evaluates the perspectives adopted when perceiving stimuli presented through sequential vibrations on the body. In particular, to interpret ambiguous tactile symbols (e.g., the letters ‘b’, ‘d’, ‘p’, and ‘q’), people can adopt different perspectives that can be either self-centred (egocentred-trunk or egocentred-head) or other-centred (i.e., decentred, see Fig. 1). Note that egocentred perspective can be equivalent to a first-person perspective also described in the literature. One's preferred perspective is first obtained when freely recognising the letters, then one’s flexibility is obtained when perspectives are imposed to measure the cost of switching between egocentred and decentred perspectives.

The aim of the present study was to investigate whether the ability to adopt and flexibly change between different spatial perspectives is influenced by conscious dimensions of interoceptive abilities, namely interoceptive accuracy, sensibility and awareness^7^. To this aim, we assessed in the same sample of neurotypical participants spatial perspective-taking with the Graphesthesia task (adapted from Arnold et al.^21^) as well as cardiac interoceptive accuracy, with the Heartbeat counting task^19^, cardiac interoceptive awareness, with the correspondence between the heartbeat counting task performance and the confidence about the heartbeat estimation^7^, and interoceptive sensibility, with a self-reported measure (MAIA-2^22^). Given the previous hypothesis of a link between high interoceptive accuracy and stronger perceived boundaries between internal states and the external world^23^, we expected an increased cost of switching between spatial perspectives to be linked to improved perception of one’s internal bodily signals (i.e. interoception).

## Methods

### Participants

99 neurotypical participants (50 females, mean age = 25.68 years, SD = 5.03) were recruited by the INSEAD-Sorbonne Université Behavioural Lab. Lack of current or past diagnosis of psychiatric, neurological, neurodevelopmental (including dyslexia) or cardiovascular conditions as well as no use of medication to treat such conditions were criteria for inclusion in the study. The sample size was determined based on prior power calculations (Cohen’s d set at 0.4; G*Power 3.1) in accordance with the average effect sizes reported in Job et al.^24^. The research was conducted after participants provided written informed consent. The research was approved by the INSEAD Institutional Review Board and was conducted in accordance with the Declaration of Helsinki. The experiment took approximately one hour to complete and the participants received monetary compensation for their time.

### Procedure and measures

The participants first underwent the heartbeat counting task^19^ and time estimation task^25^. Then, they filled out a questionnaire on interoceptive sensibility (MAIA-2^22^). They next completed the Graphesthesia task as described below. The procedure is described in Fig. 1.

#### Assessment of interoception

Interoceptive abilities were assessed in accordance with Garfinkel et al.^7^. In particular, *interoceptive accuracy* was measured with the heartbeat counting task^7,19^. While sitting comfortably in front of the computer, participant's heart rate was recorded using a heart rate oximeter (*ADInstruments*, Sydney, Australia), attached to the participant's non-dominant index finger and connected to a laptop with the LabChart software (*ADInstruments*, Sydney, Australia). Before starting the task, a 2-min baseline was recorded to measure the resting heart rate while the participants were invited to take a comfortable position and rest. During the execution of the task, the participants were not allowed to use strategies such as taking their pulse from their wrist, chest or other body parts. Instead, they were instructed to “feel” the sensation of their heart beating and to report the number of heartbeats they felt, without guessing (instructions were adapted from Desmedt et al.^25^). The participants did not receive any feedback regarding their performance. The participants then received a 30 s training test. Each trial consisted of a fixation cross, then an auditory cue instructing the participant to start counting the number of felt heartbeats, followed by a second auditory cue indicating to stop counting. Following the second auditory cue, participants were asked to report on the computer the number of heartbeats they felt. The task consisted of six different trials, with 6 different time periods (20 s, 25 s, 30 s, 35 s, 40 s, 45 s). Participants did not receive any cue about the trial duration; and trials were randomised across participants. The recorded number of heartbeats was extracted for each trial using the ‘count peaks' function from the LabChart software (*ADInstruments*, Sydney, Australia). Note that all trials were also manually checked for any missing/over counted heartbeats.

Then, accuracy of heartbeat perception was calculated as the mean score of heartbeat counting intervals according to the following formula: 1/6 Σ [(1-(|recorded heartbeats – counted heartbeats|)/recorded heartbeats))]^26^. Using this transformation, interoceptive accuracy scores could vary between 0 and 1, with higher scores indicating smaller differences between recorded and perceived heartbeats (i.e. greater accuracy corresponds to higher interoceptive accuracy).

Furthermore, participants were asked to rate their confidence (1 = not confident at all; 10 = extremely confident) in their own response accuracy after each trial.

*Interoceptive awareness* during the heartbeat counting task was calculated from trial-by-trial correlations (i.e. Pearson’s r) between accuracy and confidence in performing the heartbeat counting task. The interoceptive awareness scores can range from -1 to 1, with higher values representing higher congruency and better interoceptive awareness, and lower values referring to lower congruence and therefore worse interoceptive awareness (see Garfinkel et al.^7^).

*Interoceptive sensibility* was assessed through the translated French version of the Multidimensional Assessment of Interoceptive Awareness (MAIA-2^22^). The MAIA-2 consists of 37 items distributed in eight scales: (i) noticing, as the awareness for comfortable, uncomfortable and neutral body sensations (Cronbach’s alpha = 0.63); (ii) not-distracting, as the tendency to ignore or distract oneself from sensations of pain or discomfort (Cronbach’s alpha = 0.78), (iii) not-worrying, investigating emotional distress or worry with sensations of pain or discomfort (Cronbach’s alpha = 0.77); (iv) attention regulation, as the ability to sustain and control attention to body sensations (Cronbach’s alpha = 0.74); (v) emotional awareness, as the awareness about the connection between body sensations and emotional states (Cronbach’s alpha = 0.82), (vi) self-regulation, as the ability to regulate psychological distress by attention to body sensations (Cronbach’s alpha = 0.78), (vii) body listening, as the tendency to actively listen to body signals (Cronbach’s alpha = 0.77); (viii) trusting, as the tendency to experience one’s body as safe and trustworthy (Cronbach’s alpha = 0.80). The participants were required to rate on a 0–5 scale (0 = never, 5 = always) the frequency with which each one of the listed situations happens.

It should be underlined here that previous studies suggested that the not-distracting and the Not-Worrying factors are not associated with the other factors of the MAIA^27–29^. Moreover, only six out of eight subscales (i.e. noticing, attention regulation, emotional awareness, self-regulation, body listening, and trusting) resulted in a common general factor of interoceptive sensibility^30^. To confirm this, a confirmatory factor analysis was performed on our data set on the eight subscales scores. Results of the principal component analysis confirmed the presence of one higher order factor, consisting of six lower order domains (standardised saturations on the first factor: noticing = 0.677; attention regulation = 0.757; emotional awareness = 0.741; self-regulation = 0.752; body listening = 0.782; trusting = 0.596; variance explained: 39.23; eigenvalues: 3.139) and one separate factor consisting of two lower order domains (standardised saturations on the second factor: not-distracting = −0.571; not-worrying = 0.889; variance explained: 57.05; eigenvalues: 1.426). Therefore, in our study, the MAIA-2 interoceptive sensibility variable was computed as the sum of the six subscales addressing the first higher order factor (i.e. noticing, attention regulation, emotional awareness, self-regulation, body listening, and trusting), with higher scores corresponding to higher interoceptive sensibility.

The participants also underwent a *time estimation task*^31,32^, a control measure used to assess that participants were not just counting time during the heartbeat counting task^31^. The time estimation task consisted of counting the number of seconds in a set period of time (20 s, 30 s, 35 s; trials were randomised across participants); the number of seconds reported by each participant was compared to the actual duration of that trial. For each trial, the participants had to start counting the seconds starting from a ‘’go’’ auditory signal and stop when the “stop” acoustic signal was presented, similarly to the heartbeat counting task. The formula used to calculate interoceptive accuracy was also used to calculate the time estimation task accuracy. The heartbeat counting task and the time estimation task were designed using the Expyriment computer software^33^.

#### The graphesthesia task paradigm

The tactile stimuli were presented by means of 9 rectangular vibrators arranged in a 3-by-3 array with a centre-to-centre spacing of 5 cm (as in Arnold et al.^20^, and Job et al.^24,34^). A nine-channel amplifier drives each vibrator independently at a frequency of 250-Hz. The vibrator array was placed on the participant’s abdomen symmetrically to their body midsagittal line. Only one layer of clothing was allowed between the skin and the vibrators and the participants individually selected the intensity of each vibrator by means of a method of adjustment in order for them to perceive all the vibrators with the same intensity. The participants wore noise-reducing headphones with a noise reduction rating of 30 dB, in order to mask any sounds made by the vibrators. The letters b, d, p, and q were delivered through sequential activations of each vibrator, and each letter was presented equally often (for a detailed description, see Arnold et al.^21^).

The participants’ task was to answer which of the four letters they perceived, as quickly and as accurately as possible (from the start of the stimulation and before the 3 s time-out). The participants completed two sessions. In Session 1, the participants were free to adopt any perspective (e.g. egocentred-head, egocentred-trunk, decentred) to recognize the letters traced on their abdomen (3 blocks of 16 trials). In Session 2, a cue at the start of each trial instructed the participants which perspective to adopt on that trial. The cue changed between participants’ egocentred and decentred perspective every other trial (AABBAABB format), in order to get repeat and switch trials. However, to increase difficulty and reduce habituation and cognitive strategies, some ‘catch’ trials of other egocentred perspectives were introduced within each block (e.g. if the participants’ natural perspective was centred on their trunk, then the catch trials were egocentred-head; vice-versa, if the participants’ natural perspective was egocentred-head, then the catch trials were egocentred-trunk). Note, that after each catch trial we added a trial of non-interest used to create a switch repeat on the trial after. In session 2, the participants completed three blocks of 25 trials (with 16 trials of interest in each block, 4 catch trials, 4 trials of non-interest and 1 trial at the beginning of the block that was only used to create a switch/repeat on the second trial). If in Session 1, the participants adopted an egocentred-trunk perspective more than the other perspective, the egocentred-trunk perspective was used as the Egocentred perspective in Session 2. However, if the egocentred-head perspective was chosen more often, then the egocentred-head perspective was chosen as the Egocentred perspective in session 2. Responses were given by pressing one of four adjacent keys on a keyboard labelled b, d, p and q with the index finger of their preferred hand.

### Data analyses

There are three possible perspectives that participants can adopt when interpreting ambiguous symbols displayed on the body surface. For instance, for the letter “b” traced on the stomach, individuals adopting a egocentred-trunk perspective will report the mirror reversed letter “d”; those adopting a egocentred-head perspective will report the 180°-rotated letter “q”, and those adopting a decentred perspective, as if the letter was perceived from the perspective of an external location, will report the letter “b”. The fourth possibility, reporting a “p” would correspond to a egocentred-head perspective with a projection backward, as if the participants imagined the letter traced on their back. This possibility does not correspond to a natural perspective. In session 1, the participants were asked to report the letter as is the most natural to them, without constraints. Hence, there are no errors and the reported letters are used to compute the consistency of response in each perspective, and determine the perspective that is the most adopted by each participant. Given the three possible perspectives that can be adopted on tactile stimuli, a chance level cut-off was fixed at 33%. On the other hand, in session 2, a perspective is imposed, hence if the reported letter corresponds to this perspective, the answer is counted as correct and other letters are considered as incorrect responses. Four participants were excluded due to poor average consistency in session 1. Here, results below 33% indicate that the participants were not able to choose a natural perspective among the three possible ones. As for session 2, we excluded five participants due to poor average accuracy (below 33%) in adopting their natural perspective when imposed. The final sample consisted of 90 healthy individuals (45 females, mean age = 25.43 years, SD = 4.99, age range = 18—41). Statistical analyses were conducted using JASP (version 0.16) and SPSS (version 25) computer software.

#### The graphesthesia task

The natural perspective adopted by each participant was extracted from the perspective they adopted the most in Session 1. Two separate mixed repeated-measure ANOVAs for accuracy and reaction times (RTs) were conducted. The perspective imposed in session 2 (i.e. egocentred or decentred) and type of trial (switch or repeat) were entered as within-subject factors, and the perspective freely adopted in session 1 (i.e. 3 groups: egocentred-trunk, egocentred-head, and decentred) was entered as a between-subject factor. Correction for multiple comparisons was made using Bonferroni adjustment for all post hoc analyses of significant interactions.

#### Interoception and spatial perspective-taking

Firstly, Spearman correlations were conducted to exclude a possible relationship between interoceptive accuracy and time estimation task (see Desmedt et al.^31^), and to explore possible relationships between interoceptive measures (i.e. interoceptive accuracy, interoceptive sensibility, and interoceptive awareness; see Garfinkel et al.^7^). The α level was set to 0.01 to account for multiple comparisons. All tests were two-tailed.

A one-way MANOVA was used to check possible differences between the perspective freely adopted in session 1 (i.e. egocentred-trunk, egocentred-head, and decentred) on dimensions of interoception (i.e. interoceptive accuracy, interoceptive sensibility, interoceptive awareness).

Linear regressions were conducted to investigate whether any of the three interoceptive dimensions predicted the consistency in adopting each perspective in session 1. Specifically, three stepwise linear regression models were carried out, entering dimensions of interoception (i.e. interoceptive accuracy, interoceptive sensibility, interoceptive awareness) and performance at the time estimation task as predictors, and average consistency in each perspective freely adopted in session 1 (i.e. egocentred-trunk, egocentred-head, and decentred) as separate dependent variables.

Then, four stepwise linear regressions were conducted to investigate whether any of the three interoceptive dimensions predicted accuracy in repeating the same perspective or switching between egocentred and decentred perspectives in session 2. Specifically, dimensions of interoception (i.e. interoceptive accuracy, interoceptive sensibility, and interoceptive awareness) and performance at the time estimation task were entered as predictors, and accuracy in the different conditions of session 2 (i.e. average accuracy in: repeating egocentred trials, switching from decentred to egocentred trials, repeating decentred trials, and switching from egocentred to decentred trials) as dependent variables. Note that for all analyses in session 2 we considered both egocentred-trunk and egocentred-head as egocentred, without any distinction. Similarly, four stepwise linear regressions were conducted using average RTs instead of accuracy as dependent variables (i.e. average RTs in: repeating egocentred trials, switching from decentred to egocentred trials, repeating decentred trials, and switching from egocentred to decentred trials). For all stepwise linear regression models, a mixed method of selection was used. Similarly to the forward method, the predictor with the highest correlation with the outcome variable is entered first, but every time a predictor is added to the model, a removal test is made to constantly reassess the model by removing redundant predictors.

Note, that due to normality distribution violations and to control for outliers, we also ran bootstrap regression models with 1000 repetitions and a seed of 1, leading to the same significant results.

## Results

### The graphesthesia task

**Figure 2:**
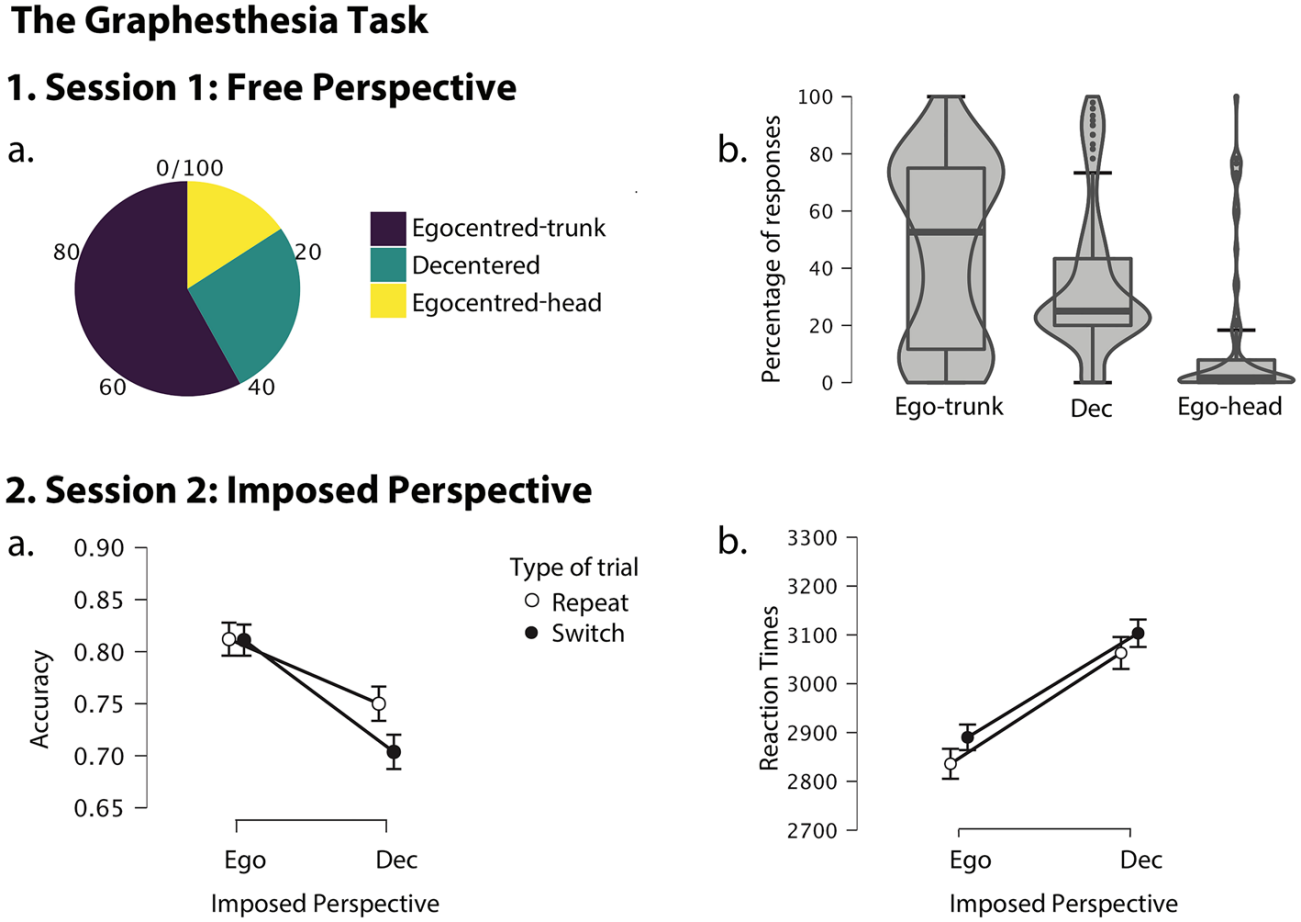
Results of the Graphesthesia task. (**1.a**) Proportions of participants assigned as egocentred-trunk, egocentred-head, or decentred based on the majority of their responses in the free session (Session 1). (**1.b**) Percentage of responses for each potential perspective (Egocentred-trunk, Decentred, Egocentred-head) within session 1 across all participants (i.e., without taking into account which perspective participants adopted the most). (**2.a**). Average accuracy and (**2.b**) Reaction Times results in Session 2 for trials where participants were repeating the same or switching between imposed perspectives, regardless of their preferred perspective. *Ego* egocentred, *Dec* decentred.

Results on the Graphesthesia task are summarised in Fig. 2.

In Session 1, 58% of participants (N = 52) adopted an egocentred-trunk perspective, 15% (N = 14) an egocentred-head perspective, and 27% (N = 24) a decentred perspective.

In Session 2, results of the repeated-measure ANOVA on accuracy showed a significant main effect of perspective imposed in session 2, *F*(1,87) = 27.29, *p* < 0.001, *η*^*2*^_*p*_ = 0.239, with higher accuracy when participants were asked to adopt an egocentred than a decentred imposed perspective (*mean difference* = 0.121, *SE* = 0.023), and a trend towards a main effect of the type of the preceding trial (repeat vs. switching perspective), *F*(1,87) = 3.478, *p* = 0.066, *η*^*2*^_*p*_ = 0.038, but no significant interaction between the imposed perspective and the type of the preceding trial (*F*(2,87) = 1.148, *p* = 0.287, *η*^*2*^_*p*_ = 0.013). In order to investigate whether the natural perspective adopted in Session 1 (free session) had an effect on the accuracy and RT in the imposed repeat-switch session, participants were split into groups depending on the perspective they adopted the most in the free session 1 (perspective group: Egocentred-trunk, Decentered and Egocentred-head). No main between-subject effect of the “perspective group” on the accuracy in session 2 was found (*F*(2,87) = 0.449, *p* = 0.640, *η*^*2*^_*p*_ = 0.010). However, an interaction between the imposed perspective in session 2 and the perspective group was found for accuracy (*F*(2,87) = 12.18, *p* < 0.001, *η*^*2*^_*p*_ = 0.219). Bonferroni-corrected post-hoc comparisons showed that for the egocentred-trunk group, recognition accuracy significantly decreased when required to report the stimuli from a decentred perspective compared to an egocentred perspective (*mean difference* = 0.069, *SE* = 0.027, *p* = 0.011, *t* = 2.59). The same was true for the egocentred-head group (*mean difference* = 0.304, *SE* = 0.051, *p* < 0.001, *t* = 5.93) but not in the decentred group (*mean difference* = −0.009, *SE* = 0.039, *p* = 0.825, *t* = −0.22). Moreover, a significant interaction for accuracy was found between the type of the preceding trial in session 2 and the perspective group (*F*(2,87) = 4.39, *p* = 0.015, *η*^*2*^_*p*_ = 0.092). Bonferroni-corrected post-hoc comparisons showed that recognition accuracy was significantly lower when the perspective instruction switched compared to when it repeated in the egocentred-trunk group (*mean difference* = 0.038, *SE* = 0.013, *p* = 0.005, *t* = 2.85) as well as in the egocentred-head group (*mean difference* = 0.054, *SE* = 0.026, *p* = 0.042, *t* = 2.06), but not in the decentred group (*mean difference* = −0.026, *SE* = 0.020, *p* = 0.193, *t* = −1.31).

Results of the repeated-measure ANOVA on RTs showed a significant main effect of imposed perspective in session 2 (*F*(1,85) = 50.75, *p* < 0.001, *η*^*2*^_*p*_ = 0.374); with higher RTs when adopting a decentred imposed perspective in comparison to an egocentred one (*mean difference* = −278.28, *SE* = 39.06). A significant main effect on RTs was also found for the type of the preceding trial (*F*(1,85) = 5.24, *p* = 0.025, *η*^*2*^_*p*_ = 0.058); with higher RTs in switch trials in comparison to repeat trials (*mean difference* = −54.27, *SE* = 23.7). No interaction was found between the type of perspective and type of trial (*F*(2,85) = 0.001, *p* = 0.970, *η*^*2*^_*p*_ < 0.001).

No between-subject effect of the perspective group on the RTs in session 2 was found (*F*(2,87) = 0.959, *p* = 0.387, *η*^*2*^_*p*_ = 0.022). However, a significant interaction between imposed perspective in session 2 and the perspective group was found on RTs (*F*(2,85) = 17.80, *p* < 0.001, η^*2*^_*p*_ = 0.295). Bonferroni-corrected post-hoc comparisons showed that RTs were significantly higher in decentred than egocentred perspective in the egocentred-trunk group (*mean difference* = 216.95, *SE* = 45.43, *p* < 0.001, *t* = −4.77), as well as in the egocentred-head group (*mean difference* = 631.25, *SE* = 85.85, *p* < 0.001, *t* = −7.35), but not in the decentred group (*mean difference* = 13.37, *SE* = 65.57, *p* = 0.839, *t* = 0.20).

### Interoception and spatial perspective-taking

Interoceptive performance accuracy for the heartbeat counting task was mean (SD) = 0.379 (0.257), range: 0–0.961; metacognitive interoceptive awareness score was mean (SD) = 0.229 (0.544), range: −0.963 to 1; the MAIA-2 interoceptive sensibility was mean (SD) = 18.37 (4.5) range: 8.13–28.01; the time estimation task performance accuracy was mean (SD) = 0.672 (0.225), range: 0.067–0.966.

Results of the Spearman correlation analysis showed no significant correlation between interoceptive accuracy and the time estimation task (*rho* = 0.130, *p* = 0.228, 95% CI [−0.124, 0.316]), confirming that participants were not just counting time during the heartbeat counting task.

Moreover, no correlation between the different dimensions of interoception reached significance with the corrected threshold (α = 0.01): (i) interoceptive accuracy and interoceptive awareness (*rho* = 0.224, *p* = 0.046, 95% CI [0.004, 0.423]); (ii) interoceptive accuracy and interoceptive sensibility (*rho* = 0.195, *p* = 0.069, 95% CI [−0.015, 0.388]); (iii) interoceptive sensibility and interoceptive awareness (*rho* = −0.024, *p* = 0.831, 95% CI [−0.243, 0.197]). Moreover, results of the one-way MANOVA showed no significant difference in any dimension of interoception based on the perspective freely adopted in session 1 (i.e. Egocentred-trunk, Decentred, Egocentred-head; *F*(3,86) = 0.475, *p* = 0.826, Wilk’s lambda = 0.963, *η*^*2*^_*p*_ = 0.019). More specifically, there was no significant effect of perspective freely adopted on interoceptive accuracy (F(1,85) = 0.637 , p = 0.532, *η*^*2*^_*p*_ = 0.016), interoceptive awareness (F(1, 85) = 0.406, p = 667, *η*^*2*^_*p*_ = 0.010), or interoceptive sensibility (F(1, 85) = 0.387, p = 0.680, *η*^*2*^_*p*_ = 0.010).

**Figure 3:**
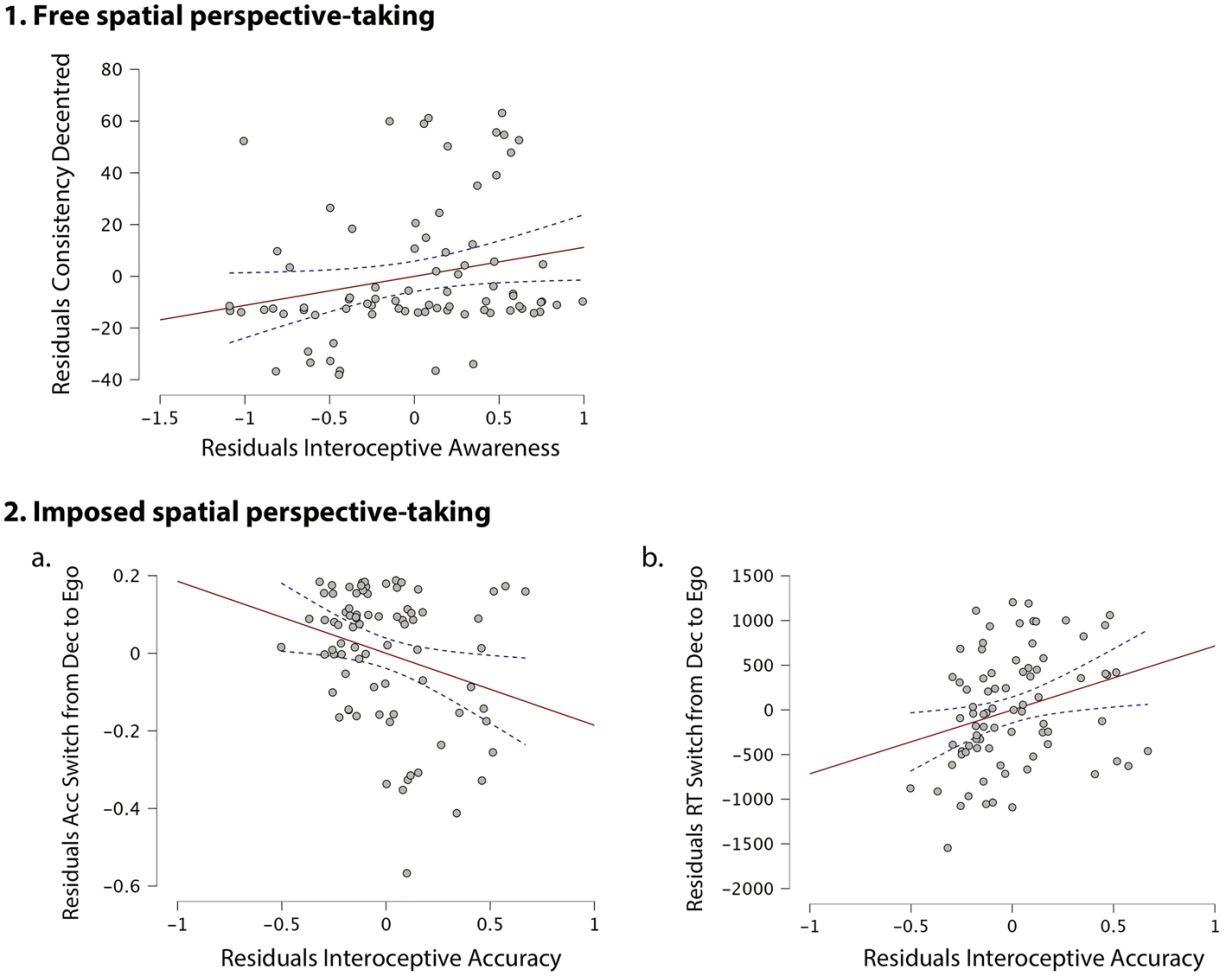
Partial correlation plots of linear regression analyses. (**1**) Residual plot of consistency on decentred trials in session 1 and interoceptive awareness. (**2.a**) Residual plot of accuracy when switching from decentred to egocentred perspectives in session 2 and interoceptive accuracy. (**2.b**) Residual plot of RTs when switching from decentred to egocentred perspectives in session 2 and interoceptive accuracy. Data points for each participant are depicted by circle markers and standard errors are depicted in the shaded intervals.

Main results on the significant linear regressions are summarised in Fig. 3. When considering interoceptive dimensions and the time estimation task as predictors of performance on the Graphesthesia task, data from the stepwise linear regressions showed that the best fitted model for the prediction of consistency on decentred trials was significant (*F*(1,79) = 4.627, *p* = 0.035, *R* = 0.237, *R*^*2*^ = 0.056), with the only predictor being interoceptive awareness. Indeed, higher interoceptive awareness significantly predicted higher consistency on decentred trials in session 1 (β = 0.237, *t* = 2.151, *p* = 0.035, *VIF* = 1). The bootstrap regression model confirmed the same significant result (β = 0.235, *t* = 2.036, *p* = 0.045). The best fitted model for the prediction of consistency on head trials was significant (*F*(1,79) = 5.254, *p* = 0.025, *R* = 0.251, *R*^*2*^ = 0.063), with the only predictor being the time estimation task. Indeed, higher performance in the time estimation task significantly predicted lower consistency on head trials in session 1 (β = −0.251, *t* = −2.292, *p* = 0.025, *VIF* = 1). However, the bootstrap regression model showed only a trend towards significance for the time estimation task in predicting consistency on head trials (β = −0.259, *t* = −2.291, *p* = 0.054). The linear regression model for the prediction of consistency in trunk perspectives adopted in session 1 on any interoceptive dimension or time estimation did not reach significance.

As for accuracy in switching between imposed perspectives on the Graphesthesia task in session 2, the best fitted model for the prediction of accuracy in switching from decentred to egocentred trials in session 2 was significant (*F*(2,79) = 6,159, *p* = 0.015, *R* = 0.271, *R*^*2*^ = 0.073), with the only predictor being interoceptive accuracy. Specifically, higher interoceptive accuracy predicted lower accuracy when switching from a decentred to an egocentred imposed perspective (β = –0.271; *t* = −2.482, *p* = 0.015, *VIF* = 1). The bootstrap regression model confirmed the same significant result (β = −0.300, *t* = −2.584, *p* = 0.025). No other linear regression model for the prediction of accuracy in session 2 on any interoceptive dimension reached significance.

As for RTs when switching between imposed perspectives on the Graphesthesia task in session 2, results showed that the best fitted model for the prediction of RTs when switching from decentred to egocentred trials in session 2 was significant (*F*(2,79) = 6.424, *p* = 0.003, *R* = 0.378, *R*^*2*^ = 0.143), with predictors being interoceptive accuracy and time estimation task. Specifically, higher interoceptive accuracy predicted slower RTs (β = 0.337, *t* = 3.15, *p* = 0.002, *VIF* = 1.029), and higher performance in the time estimation task predicted faster RTs (β = −0.237, *t* = −2.215, *p* = 0.030, *VIF* = 1.029) when returning to an egocentred perspective from a decentred one. The bootstrap regression model confirmed the same significant result for interoceptive accuracy (β = 0.334, *t* = 2.976, *p* = 0.011) and a trend towards significance for the time estimation task (β = −0.238, *t* = −2.186, *p* = 0.050). The best fitted model for the prediction of RTs when repeating decentred trials in session 2 was significant (*F*(1,77) = 6.08, *p* = 0.016, *R* = 0.272, *R*^*2*^ = 0.074), with the only predictor being time estimation task. Specifically, higher performance in the time estimation task predicted faster RTs (β = −0.272, *t* = −2.466, *p* = 0.016, *VIF* = 1) when repeating decentred trials. The bootstrap regression model confirmed the same significant result (β = −0.300, *t* = −2.677, *p* = 0.003). No other linear regression model for the prediction of RTs in session 2 on any interoceptive dimension was significant.

## Discussion

The present study investigated whether the natural adoption of an egocentred versus a decentred perspective and the ability to switch between different spatial perspectives is predicted by multiple dimensions of interoception (interoceptive accuracy, sensibility or awareness). Spatial perspective-taking was assessed with a new version of the tactile Graphesthesia task to better assess flexibility in changing spatial perspectives.

In session 1 of the Graphesthesia Task, in which the participants were free to adopt any perspective (e.g. egocentred-head, egocentred-trunk, decentred) to recognize the letters traced on their abdomen, the majority of them adopted an egocentred-trunk (58%), followed by a decentred (27%) and an egocentred-head (15%) perspective. When the perspective was imposed in session 2, the participants were more accurate and faster when asked to adopt an egocentred than a decentred perspective. Moreover, in the group of participants who freely adopted an egocentred perspective in session 1 (i.e. egocentred-trunk and egocentred-head groups), recognition accuracy decreased and RTs increased when required to report the stimuli from a decentred perspective compared to an egocentred perspective. As for the type of preceding trial, RTs were slower when the perspective instruction switched compared to when it repeated and recognition accuracy was lower in switch trials compared to repeat trials for egocentred-trunk and egocentred-head groups. This result confirms the idea of a natural, spontaneous egocentred perspective in these participants, and replicates the cost of switching observed previously^21^.

Regarding the influence of interoceptive abilities on the spontaneous adoption of different spatial perspectives, the present study shows that higher cardiac metacognitive interoceptive awareness, rather than behavioural performance on cardiac interoceptive accuracy or subjective interoceptive sensibility, predicts a higher consistency in adopting a decentred perspective. This result suggests that the more individuals are aware of their own cardiac activity, the more likely they are to spontaneously adopt a decentred spatial perspective. To date, this is the first study investigating the role of metacognitive awareness about cardiac signals (i.e. cardiac interoceptive awareness) in spontaneously adopting different spatial perspectives. This opens future investigations exploring deeper this relationship. Why are participants with higher interoceptive awareness more prone to adopting a decentred perspective? Is this due to the fact that once we are aware of our body signals, we can become detached from oneself and take the other/external point of view? This would be in line with the theory suggested by Fotopoulou & Tsakiris^35^, along which interoceptive awareness is part of the cognitive acquisitions that allow us to progressively solidify self-other distinctions, as well as to understand and empathise with others. Our results extend this idea to the spatial domain, revealing that metacognitive awareness of cardiac signals facilitates taking another’s spatial-perspective.

Regarding the influence of interoceptive abilities on switching between different spatial perspectives, higher cardiac interoceptive accuracy was found to predict lower accuracy and slower reaction times when switching from a decentred to an egocentred perspective. In other words, participants with higher interoceptive accuracy experienced more difficulty in switching back to an egocentred perspective. Reduced flexibility in perspective taking may be related to a sharper perceived boundary between the self and the external world. Research on bodily illusions—as a probe of the plasticity of the boundaries of the bodily self—has shown that individuals with higher cardiac interoceptive sensitivity are less susceptible to the rubber hand illusion^36^, in which an illusory sense of ownership over a rubber hand is induced by synchronous visual-tactile stimulation. This could reflect the idea that individuals with higher interoceptive accuracy possess a less malleable sense of self^37,38^. However, it is interesting to note that high interoceptive accuracy in our study was only related to a difficulty with switching from a decentred perspective to one's own (egocentred) perspective, rather than switching away from one's own perspective to a decentred one.

We acknowledge that the validity of interoceptive accuracy, assessed with the heartbeat counting task, has been debated in recent years^25,39^. In particular, some confounding factors (e.g. common knowledge of typical cardiac frequency and time estimation) and the type of instructions given to participants could affect the reliability of the heartbeat counting task. To overcome these issues, following Desmedt et al.^25^, instructions were adapted to reduce the contribution of estimation-based strategies in the heartbeat counting performance. Moreover, a time estimation task was administered to the participants and was not found to be associated with performance on the heartbeat counting task, demonstrating that the participants were not just counting time during the task^31^. However, we did not take into account the presence of other variables, such as acquired knowledge of heart rate at rest^31,40^ and levels of body fat^41^. Moreover, other criticisms of the heartbeat counting task have been recently highlighted. For example, the use of pulse oximeters to measure heartbeats has been shown to be associated with higher performance on the heartbeat counting task when compared to a classic ECG, due to the greater pressure of the oximeter on the participant's skin which increased the contact between the skin receptors and blood^41,42^. To limit this effect, participants were asked whether they could feel their pulse before starting the task. If it was the case, we made sure to loosen the oximeter. However, future research should replicate the present results by using an ECG as well as another task assessing cardiac interoceptive abilities (e.g. Cardiac Discrimination task). In addition, it would be interesting in future research to assess whether our results on the links between cardiac interoceptive abilities and perspective taking generalize to other interoceptive modalities (e.g., gastric, hunger, temperature). Our results contrast with those of Erle^18^, where a more accurate and faster performance in a visual perspective-taking task was related to higher interoceptive accuracy. This could be due to the different sensory channels involved in the two tasks. Indeed, during the Graphesthesia task participants did not have to visualise a situation on the computer screen but they had to feel a tactile vibration on the abdomen. Perceiving tactile information is thought to be part of the construct of interoception itself^43^ and it is conceivable that this kind of task involves more embodied mechanisms^44,45^. This could also be due to the fact that the Graphesthesia task was designed to tackle the flexibility to change between tactile-spatial perspectives with no explicit other person; while in the Level 2 VPT task used by Erle^18^, the participant always had to take the avatar’s perspective (sometimes congruent or not with their own egocentric visual point of view). To disentangle between the effect of sensory modalities and the effect of presence versus absence of others, future studies should contrast, with a similar paradigm, tactile and visual perspective-taking with the position to adopt being represented or not by another person.

Moreover, research on non-spatial perspective-taking (i.e. affective and cognitive perspective-taking), showed that individuals with higher interoceptive accuracy are less prone to readily switch to another person's emotional perspective given their tendency to not blur self-other boundaries^23^. However, other results on affective and cognitive perspective-taking indicated that individuals who are more accurate about their own cardiac activity can better judge oneself and others' emotional states^15,32^. This pattern of behaviour can be affected by context, for example, in a neutral context, participants with higher interoceptive accuracy seem better at judging another's emotional state. The opposite pattern occurs in contexts of heightened autonomic state^46^. For this reason, the influence of contextual factors should also be taken into account for studying the role of interoception in spatial perspective-taking tasks in future research. It is worth noting that in the present study performance on the time estimation task was a predictor of faster reaction times when repeating decentred trials in session 2 of the Graphesthesia Task. Time estimation is a relevant mechanism for the interaction with the external environment as fundamental for attention and spatial reasoning^47,48^. More generally, cognitive estimation tasks involve several resources related to attention, memory, processing load, imagery, abstract reasoning, judgment, and decision-making^49^. Although this would go beyond the interoceptive level, it would be interesting in future studies to further investigate the role of time estimation as a skill that predicts better performance in several cognitive abilities, such as spatial perspective-taking.

In the present study only conscious dimensions of cardiac interoception were considered. Future studies should investigate how implicit aspects of bodily signals differ from interoception in their relationship to spatial perspective-taking. Indeed, neurophysiological evidence supports the hypothesis that the brain’s mapping of the internal body, but not interoceptive sensitivity, is specifically related to self-consciousness^50^ and this could also play a role in flexibly changing different points of view. Finally, the increased cost of switching between spatial perspectives in individuals with higher interoceptive abilities could be influenced by the allocation of attentional resources depending on task demands^51^. Future studies should directly test this hypothesis with an additional task of attention towards interoceptive compared to exteroceptive input. Taken together, our results provide new insights about the role of interoceptive processing in spatial perspective-taking, seen as two embodied processes. In particular, interoceptive abilities were shown to influence where people place themselves to perceive tactile stimuli, either according to an egocentred (or first person) perspective or from a decentred (or third-person) perspective. Interoceptive abilities also influence the ability to switch between these perspectives. These results thus provide insight about the factors influencing how we juggle two requirements: perceiving according to an egocentred perspective, which is crucial to integrating different stimuli across sensory modalities, and hence central for the unity of the self and perceiving according to a decentred perspective, which is necessary for understanding external space and communicating spatial knowledge with others.

## Data Availability

De-identified data for the present experiment used in the results section is available on the Open Science Framework and on request to the authors (https://osf.io/c76er/?view_only=1ad77ba98fe345b89efcb70d77ced9cf).
